# Prognostic value of gut microbiota and low-density lipoprotein cholesterol subfractions in patients with ST-segment elevation myocardial infarction

**DOI:** 10.3389/fimmu.2025.1610001

**Published:** 2025-08-13

**Authors:** Siliang Xia, Yun Liu, Mengzhu Wang, Dandan Liu, Xiaobing Zhang, Ling Lin, Ming Wen, Shushen Ji, Jiaying Li, Xiangming Zhang, Huihui Jiang

**Affiliations:** ^1^ Department of Cardiology, Nanjing Jiangbei Hospital, Nanjing, Jiangsu, China; ^2^ Zhangjiang Center for Translational Medicine, Shanghai Biotecan Pharmaceuticals Co., Ltd., Shanghai, China; ^3^ Teaching Research Department, Changji Branch of the First Affiliated Hospital of Xinjiang Medical University, Changji, Xinjiang, China

**Keywords:** ST-segment elevation myocardial infarction, gut microbiota, LDL-C subfractions, interaction, risk factors

## Abstract

**Objective:**

Gut dysbiosis and the distribution of low-density lipoprotein cholesterol (LDL-C) subfractions have been implicated in cardiovascular risk among patients with ST-segment elevation myocardial infarction (STEMI). However, the prognostic significance of LDL-C subfractions in relation to gut microbiota composition remains largely unexplored. This study aimed to assess differences in gut microbiota profiles and LDL-C subfraction distribution between patients with STEMI with and without major adverse cardiovascular events (MACEs) and to elucidate their potential interplay.

**Methods:**

We enrolled 32 male population without coronary heart disease and 66 male patients with STEMI. Fecal samples were analyzed via 16S rDNA gene sequencing to assess gut microbiota diversity and composition. Plasma LDL-C subfractions were quantified using the Quantimetrix Lipoprint LDL System.

**Results:**

Among these 66 STEMI patients, 18 experienced MACEs during a median follow-up of 13 months (MACEs group), while 18 age-matched event-free patients were selected as controls (Non-MACEs group). Significant differences in gut microbiota composition, but not diversity, were observed between the two groups, with the Non-MACEs group exhibiting a greater number of marker genera. Although no significant differences were found in LDL-C subfractions between groups, multiple significant negative correlations were identified between gut microbiota and LDL-C subfractions in the MACEs group, including between *Coprococcus* and LDLC-4 (ρ=-0.5488, P<0.05), between *Coprococcus* and LDLC-5 (ρ=-0.6418, P<0.01), between *Coprococcus* and LDLC-6 (ρ=-0.4988, P<0.05), between *UCG-002* and LDLC-4 (ρ=-0.4948, P<0.05), and between *Christensenellaceae_R-7_group* and LDLC-4 (ρ=-0.5032, P<0.05). Furthermore, gut microbiota markers demonstrated superior predictive performance for MACEs compared to LDL-C subfractions, with *UCG-002*, *Christensenellaceae_R-7_group*, and *NK4A214_group* achieving AUC values >0.75.

**Conclusion:**

Gut microbiota, particularly *UCG-002*, *Christensenellaceae_R-7_group*, and *NK4A214_group*, exhibit greater prognostic potential for MACEs than LDL-C subfractions. These findings highlight the role of gut microbiota in post-STEMI risk stratification, underscoring its potential as a target for future cardiovascular interventions.

## 1 Introduction

ST-segment elevation myocardial infarction (STEMI) remains a major cause of morbidity and mortality worldwide, necessitating a better understanding of its pathophysiological mechanisms and prognostic markers (1, 2). Despite advancements in acute management, including timely reperfusion therapy and pharmacological interventions, a substantial proportion of STEMI patients develop major adverse cardiovascular events (MACEs), such as recurrent myocardial infarction, heart failure, and cardiovascular death (3–5). Identifying novel prognostic factors is essential for improving risk stratification and tailoring secondary prevention strategies. Recent studies have highlighted the gut microbiota and lipid metabolism as critical contributors to cardiovascular disease progression (6–9), yet their prognostic significance in STEMI remains underexplored.

The gut microbiota plays a pivotal role in cardiovascular health, influencing systemic inflammation, lipid metabolism, and immune responses (10, 11). Emerging evidence suggests that dysbiosis, an imbalance in gut microbial composition, is linked to the development and progression of atherosclerosis and acute coronary syndromes (ACS) (12–15). Specifically, gut-derived metabolites, such as trimethylamine N-oxide (TMAO), have been implicated in endothelial dysfunction, platelet hyperreactivity, and pro-inflammatory signaling, all of which contribute to atherothrombosis (16, 17). Additionally, alterations in gut microbial diversity have been associated with systemic metabolic disturbances, including dyslipidemia and insulin resistance (18–21), further predisposing individuals to adverse cardiovascular outcomes. However, whether gut microbiota composition differs between patients with STEMI with and without MACEs and its potential prognostic value remains unclear.

Lipid metabolism, particularly LDL-C and its particles, is a well-established determinant of atherosclerotic cardiovascular disease (ASCVD) (22, 23). While total LDL-C levels serve as a primary target for lipid-lowering therapies, growing evidence suggests that LDL-C subfractions, such as small, dense LDL particles, exhibit enhanced atherogenic potential (22, 24, 25). Small, dense LDL is more susceptible to oxidation, has a prolonged plasma half-life, and demonstrates a greater propensity for arterial wall penetration, thereby accelerating atherosclerosis progression (26, 27). Moreover, recent studies indicate that gut microbiota may modulate lipid metabolism by influencing bile acid metabolism and cholesterol homeostasis (28–30). This bidirectional interaction suggests a potential link between gut dysbiosis, LDL-C subfractions distribution, and cardiovascular risk in STEMI patients. Nonetheless, the prognostic implications of LDL-C subfractions in relation to gut microbiota composition remain largely unexplored in this patient population. Given the emerging interplay between gut microbiota, lipid metabolism, and cardiovascular disease, we hypothesize that alterations in gut microbial composition and the distribution of LDL-C subfractions may serve as prognostic markers in patients with STEMI. This study aimed to investigate the differences in gut microbiota profiles and the distribution of LDL-C subfractions between patients with STEMI with and without MACEs, and elucidated the interplay between gut microbiota and LDL-C subfractions in these two groups, which might provide novel insights into the pathophysiology of STEMI and help identify potential therapeutic targets for modulating microbial and lipidomic profiles to improve cardiovascular outcomes.

## 2 Materials and methods

### 2.1 Study design and subjects

**Figure 1:**
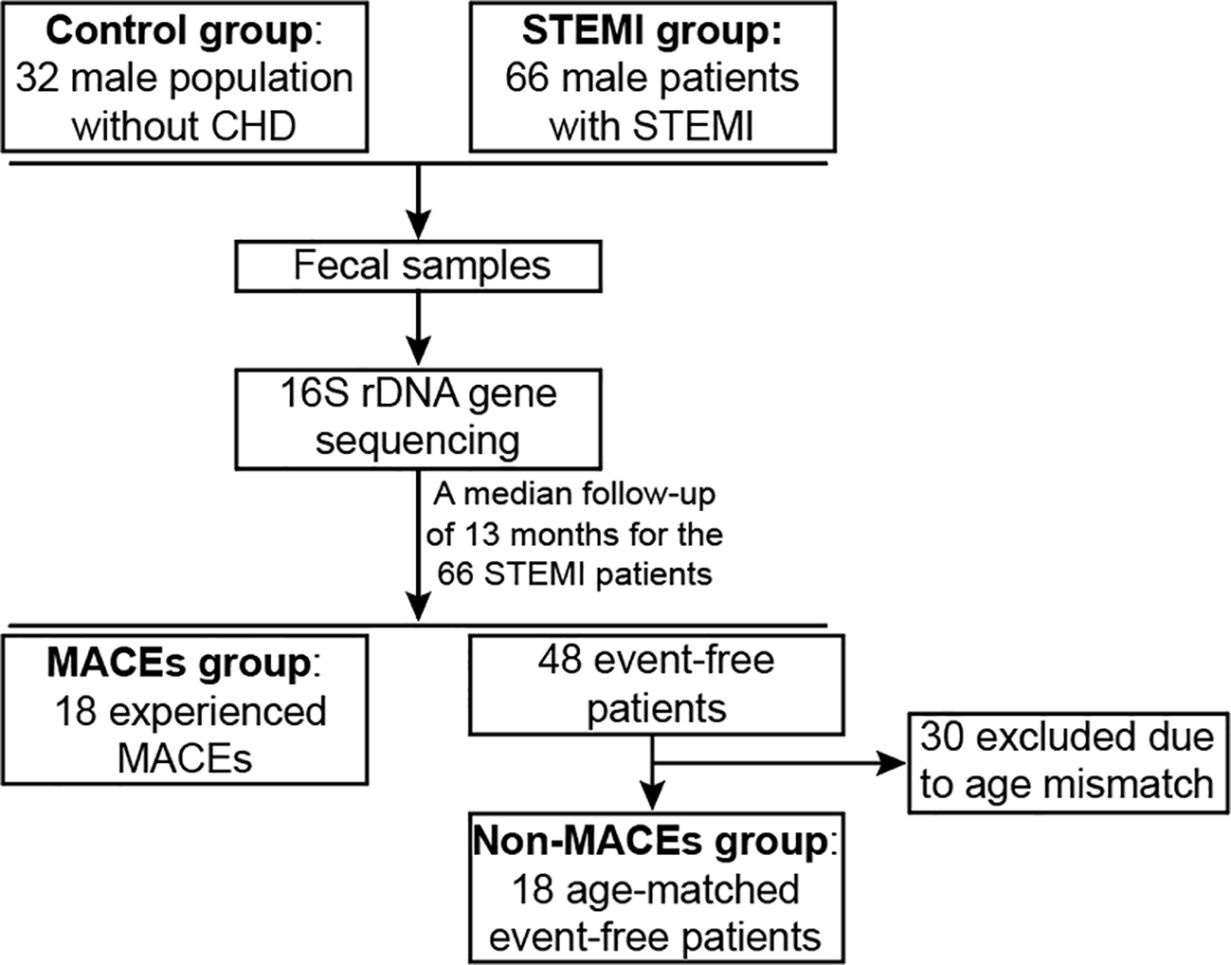
Flowchart of the study design. Thirty-two male population without CHD (Control group) and 66 male patients with STEMI (STEMI group) were enrolled in this study. Fecal samples were acquired from every individual and analyzed via 16S rDNA gene sequencing to assess gut microbiota diversity and composition. Among the 66 STEMI patients, 18 experienced MACEs during a median follow-up of 13 months (MACEs group), while 18 age-matched event-free patients were selected as controls (Non-MACEs group). CHD, coronary heart disease; STEMI, ST-segment elevation myocardial infarction; MACEs, major adverse cardiovascular events.

This case-control study comprised 32 male individuals without coronary heart disease (referred to as the Control group) and 66 male patients diagnosed with STEMI (referred to as the STEMI group) (**Figure 1**). A range of individual characteristics, including age, height, weight, risk factors, blood biochemical parameters, medications administered during hospitalization, and coronary angiography findings, were collected. The body mass index (BMI) was calculated by dividing weight (in kilograms) by the square of height (in meters). Inclusion criteria for the STEMI group included: (i) cardiac troponin (cTn) I/T levels exceeding the upper normal reference value, or creatine kinase isoenzymes exceeding the normal reference limit; (ii) ST-segment elevation observed in two or more contiguous leads on the electrocardiogram (ECG); (iii) and one or more of the following: persistent ischemic chest pain, abnormal wall motion on the ECG, and abnormal coronary angiography findings. Exclusion criteria for participants were: (i) other identifiable causes of coronary thrombosis (e.g., coronary vasospasm or systemic thromboembolism); (ii) evident active infection during hospitalization; (iii) prior history of organic digestive system or gastrointestinal surgery; (iv) history of kidney or respiratory diseases; (v) infection within one month before the study or use of probiotics, prebiotics, postbiotics, antacids, antibiotics, or related preparations. Control subjects were recruited voluntarily from individuals undergoing routine health check-ups at the same institution, all of whom had normal results. The study was approved by the Ethics Committee of Nanjing Jiangbei Hospital (#2022031). All participants provided written informed consent in accordance with the principles outlined in the Declaration of Helsinki. Additionally, this study is purely observational, and no clinical trial registration was conducted.

### 2.2 Collection of fecal samples

Fecal samples, approximately the size of two soybean grains, were collected from each participant, either by themselves or by their family members, within 3 minutes of defecation while in the hospital. After collection, the samples were placed in fecal samplers (Biotecan, Shanghai, China), sealed, labeled, and transported to Biotecan Laboratories within 2 days, ensuring the temperature remained below 18°C. Upon arrival, the samples were promptly stored at -80°C.

### 2.3 16S rDNA gene sequencing, data processing, and bioinformatics analysis

A total of 98 fecal samples (32 from the control group and 66 from the STEMI group) were collected in fecal samplers and stored at -80°C until they were processed for high-throughput sequencing. Genomic DNA was extracted from the fecal samples using the QIAamp PowerFecal Pro DNA Kit (QIAGEN, Germany). The concentration and integrity of the extracted DNA were evaluated using a NanoDrop ND-1000 spectrophotometer (Thermo Fisher Scientific, Waltham, MA, USA) and agarose gel electrophoresis, respectively. The V3-V4 hypervariable region of the bacterial 16S rRNA gene was amplified by PCR using the primer pair 341F (5′-CCTACGGGNGGCWGCAG-3′) and 805R (5′-GACTACHVGGGTATCTAATCC-3′). Sample-specific paired-end 6-bp barcodes were integrated into TruSeq adapters for multiplexed sequencing. The PCR reaction mixture included 25 μL of Phusion High-Fidelity PCR Master Mix (New England Biolabs, MA, USA), 3 μL of each 10 μM forward and reverse primer, 10 μL of template DNA, 3 μL of DMSO, and 6 μL of nuclease-free water. The thermal cycling protocol comprised an initial denaturation at 98 °C for 30 seconds, followed by 25 cycles of 98 °C for 15 seconds, 58 °C for 15 seconds, and 72 °C for 15 seconds, with a final extension at 72 °C for 1 minute. PCR products were purified using Agencourt AMPure XP Beads (Beckman Coulter, Indianapolis, IN) and quantified with the PicoGreen dsDNA Assay Kit (Invitrogen, Carlsbad, CA, USA). Following individual quantification, the amplicons were pooled in equal amounts for paired-end sequencing (2 × 250 bp) on the Illumina NovaSeq 6000 platform, conducted by Shanghai Biotecan Pharmaceuticals Co., Ltd. (Shanghai, China).

To analyze the sequencing data, the Quantitative Insights Into Microbial Ecology 2 (QIIME 2, v2017.6.0) pipeline and predefined criteria were employed (31, 32). Paired-end reads were assembled using Vsearch V2.4.4 (33). Operational taxonomic units (OTUs) were defined based on 16S rDNA gene sequences, applying a 97% similarity threshold and referencing the Greengenes database via Vsearch V2.4.4. OTUs representing less than 0.001% of the total sequences were excluded from the analysis. The final OTU table was generated by averaging, rounding, and rarefying, using 100 evenly resampled OTU subsets at 90% of the minimum sequencing depth. Abundance curves were plotted at the OTU level, and sequencing depth was evaluated and confirmed through rarefaction analysis. Alpha diversity was assessed using the Chao1, Simpson, and Shannon indices within the QIIME 2 platform. Statistical comparisons were conducted using the Pairwise Wilcox test. Beta diversity was evaluated using Weighted UniFrac principal component analysis (PCoA). To compare the intestinal bacterial composition and structure between the control and STEMI groups, Permutational Multivariate Analysis of Variance (PERMANOVA) was applied. The comparability between groups was assessed using One-way Analysis of Similarities (ANOSIM). Linear Discriminant Analysis Effect Size (LEfSe) was used to identify taxa with significantly different abundances between the groups, based on default parameters (logarithmic LDA score ≥ 2) (34, 35). The phylogenetic tree was visualized using GraPhlAn (http://huttenhower.sph.harvard.edu/GraPhlAn). To predict gut microbial functions, the Phylogenetic Investigation of Communities by Reconstruction of Unobserved States (PICRUSt, PICRUSt2 v2.3.0-b) was employed (36, 37), with the Univariate Test used to assess significant differences.

### 2.4 Sample collection and laboratory indices detection

Blood samples were obtained from all participants after an overnight fast, prior to undergoing coronary angiography and receiving any concomitant medications. The samples were then analyzed in the Department of Clinical Laboratory for routine biomarkers, including total cholesterol (TC), triglycerides (TG), high-density lipoprotein cholesterol (HDL-C), low-density lipoprotein cholesterol (LDL-C), serum creatinine, and other relevant indices. Plasma was immediately separated and subjected to centrifugation at 800×g for 10 minutes at 4°C. The LDL-C subfractions were classified and quantified using the Quantimetrix Lipoprint LDL System (Quantimetrix Corporation, Redondo Beach, CA, USA), following the manufacturer’s protocol (38). Briefly, plasma mixed with liquid loading gel was applied to the top of pre-cast 3% polyacrylamide gel tubes. After 30 minutes of photopolymerization at room temperature, the samples were electrophoresed for 1 hour, and densitometric analysis was performed at 610 nm.

### 2.5 Clinical endpoints

The primary endpoint of the follow-up was the occurrence of the first MACEs, which encompassed cardiovascular death, non-fatal ischemic stroke, recurrent myocardial infarction, the need for emergency or repeated revascularization, and rehospitalization due to heart failure, as previously defined (39–41).

### 2.6 Statistical analyses

Data analysis was performed using SPSS version 23.0 (IBM, Chicago, IL, USA) and GraphPad Prism version 7.0 (GraphPad, San Diego, CA, USA). Categorical variables were compared between groups using the chi-square test or Fisher’s exact test. For continuous variables, the Shapiro-Wilk test was applied to assess the normality of the data distribution. Comparisons between groups were made using a two-tailed Student’s t-test for normally distributed data and a Mann-Whitney U-test for non-normally distributed data. The exact number of patients and the values of continuous variables for each group are provided in the figure legends. Venn diagrams, heatmaps, and Spearman’s rank correlation were generated using R software (v3.6.3). A p-value < 0.05 was considered statistically significant in all analyses.

## 3 Results

### 3.1 Characteristics of study participants

The baseline characteristics of the Control and STEMI groups were presented in **Table 1**. When compared to the Control group, the STEMI group exhibited significantly elevated fasting plasma glucose (FPG) levels (P=0.0068) and significantly reduced high-density lipoprotein cholesterol (HDL-C) levels (P=0.0065). Moreover, the prevalence of smoking was notably higher in the STEMI group than in the Control group (P<0.0001).

**Table 1:** Baseline characteristics of controls and patients with STEMI.

Variables	Control (n = 32)	STEMI (n = 66)	P value
Age, y	59.50 (55.00, 68.75)	57.50 (48.75, 67.25)	0.1304
Height, cm	170.0 (167.3, 174.5)	172.0 (170.0, 175.0)	0.3053
Weight, kg	72.50 (67.00, 79.50)	73.50 (67.00, 80.00)	0.9113
BMI	24.98 (23.43, 28.28)	24.69 (23.55, 26.67)	0.425
Risk factors			
Hypertension, n			
Yes	14	37	0.253
No	18	29	
Diabetes mellitus, n			
Yes	2	10	0.351309
No	30	56	
Smoking, n			
Yes	4	46	0.00000034659
No	28	20	
Drinking, n			
Yes	4	22	0.051576
No	28	44	
Blood biochemical tests			
TC, mmol/L	4.565 (4.060, 4.930)	4.520 (3.878, 5.423)	0.3562
TG, mmol/L	1.245 (1.025, 1.930)	1.420 (1.170, 2.230)	0.1158
HDL-C, mmol/L	1.150 (0.9900, 1.453)	0.9850 (0.8175, 1.170)	0.0065
LDL-C, mmol/L	2.740 (2.245, 3.180)	2.820 (2.143, 3.305)	0.4406
FPG, mmol/L	5.315 (4.913, 6.035)	5.980 (5.420, 7.698)	0.0068
Creatinine	77.50 (68.25, 84.75)	78.00 (63.00, 90.50)	0.8343
Medications in hospital			
Statins, n			
Yes	0	64	8.52E-24
No	32	2	
Aspirin, n			
Yes	0	64	8.52E-24
No	32	2	
β-Blockers, n			
Yes	0	63	9.94E-23
No	32	3	
ACE inhibitors/ARB, n			
Yes	0	18	0.002776
No	32	48	
P2Y12 receptor inhibitors, n			
Yes	0	66	1.52E-26
No	32	0	
Proton pump inhibitor, n			
Yes	0	64	8.52E-24
No	32	2	
Angiotensin antagonists, n			
Yes	0	36	1.16E-08
No	32	30	
Coronary angiography features			
Number of diseased vessels, n			
1 vessel	3	26	1.55E-17
2 vessels	1	27	
3 vessels	0	13	
Mutivessel disease	0	0	
None	28	0	
AHA (B2/C), n			
Yes	0	2	1
No	32	64	
Calcified lesion, n			
Yes	0	0	Not appliable
No	32	66	
thrombus, n			
Yes	0	1	1
No	32	65	

Over a median follow-up period of 13 months (range: 12–22 months), a total of 18 patients experienced MACEs, comprising 0 cardiovascular deaths, 3 non-fatal ischemic strokes, 1 recurrent myocardial infarction, 3 cases requiring either emergency or elective repeat revascularization, 11 hospital readmissions due to heart failure, and 8 readmissions for unstable angina (**Figure 1**). When comparing patients with STEMI who developed MACEs (MACEs group) with those who remained event-free (Non-MACEs group), the MACEs group exhibited significantly lower BMI (P=0.0066) and a markedly higher CK-MB level (P=0.0349) (**Table 2**). Additionally, NT-proBNP levels were noticeably higher in the MACEs group than in the Non-MACEs group (P=0.0731).

**Table 2:** Baseline characteristics of patients with and without MACEs.

Variables	MACEs (n = 18)	Non-MACEs (n = 18)	P value
Age, y	63.50 (57.50, 74.25)	65.00 (57.75, 69.25)	> 0.9999
Height, cm	172.5 (170.0, 175.0)	170.0 (168.0, 175.0)	0.4687
Weight, kg	68.50 (64.50, 75.50)	75.00 (70.00, 80.00)	0.0576
BMI	23.58 (22.08, 25.30)	25.83 (24.22, 27.23)	0.0066
Risk factors			
Hypertension, n			
Yes	13	12	1
No	5	6	
Diabetes mellitus, n			
Yes	3	5	0.690565
No	15	13	
Smoking, n			
Yes	12	10	0.733222
No	6	8	
Drinking, n			
Yes	6	3	0.443017
No	12	15	
Blood biochemical tests			
TC, mmol/L	4.640 (3.875, 5.688)	4.445 (3.575, 5.360)	0.5608
TG, mmol/L	2.050 (1.243, 2.328)	1.285 (1.105, 2.095)	0.1997
HDL-C, mmol/L	0.9650 (0.8550, 1.193)	0.9100 (0.7700, 1.265)	0.7486
LDL-C, mmol/L	2.755 (2.075, 3.265)	2.685 (1.993, 3.505)	0.9066
FPG, mmol/L	6.675 (5.605, 9.048)	5.665 (5.415, 6.940)	0.2967
Creatinine	86.50 (68.75, 96.75)	78.00 (64.50, 92.00)	0.6331
hsCRP, mg/L	2.645 (0.9050, 5.655)	2.215 (0.7200, 8.120)	0.9439
CK-MB	22.13 (16.67, 40.25)	13.97 (7.253, 23.00)	0.0349
cTnI, μg/L	2.050 (1.790, 3.175)	1.930 (0.9050, 2.470)	0.2053
cTnT, μg/L	3.250 (0.3808, 7.518)	1.150 (0.1775, 3.748)	0.1634
LDH	604.0 (436.3, 933.8)	435.5 (243.3, 830.8)	0.1063
AST	248.5 (102.3, 356.8)	138.5 (49.25, 326.5)	0.2232
NT-proBNP, pg/mL	1076 (591.0, 1957)	721.5 (149.0, 1105)	0.0731
Medications in hospital			
Statins, n			
Yes	16	18	0.485714
No	2	0	
Aspirin, n			
Yes	16	18	0.485714
No	2	0	
β-Blockers, n			
Yes	18	16	0.485714
No	0	2	
ACE inhibitors/ARB, n			
Yes	5	3	0.690565
No	13	15	
P2Y12 receptor inhibitors, n			
Yes	18	18	Not appliable
No	0	0	
Proton pump inhibitor, n			
Yes	18	17	1
No	0	1	
Angiotensin antagonists, n			
Yes	11	14	0.470523
No	7	4	
Coronary angiography features			
Number of diseased vessels, n			
1 vessel	8	4	0.230693
2 vessels	7	7	
3 vessels	3	7	
Mutivessel disease	0	0	
AHA (B2/C)			
Yes	0	1	1
No	18	17	
Calcified lesion, n			
Yes	0	0	Not appliable
No	18	18	
thrombus, n			
Yes	0	0	Not appliable
No	18	18	

### 3.2 Differences in diversity, composition, and metabolic pathways of gut microbiota between the control group and the STEMI group

**Figure 2:**
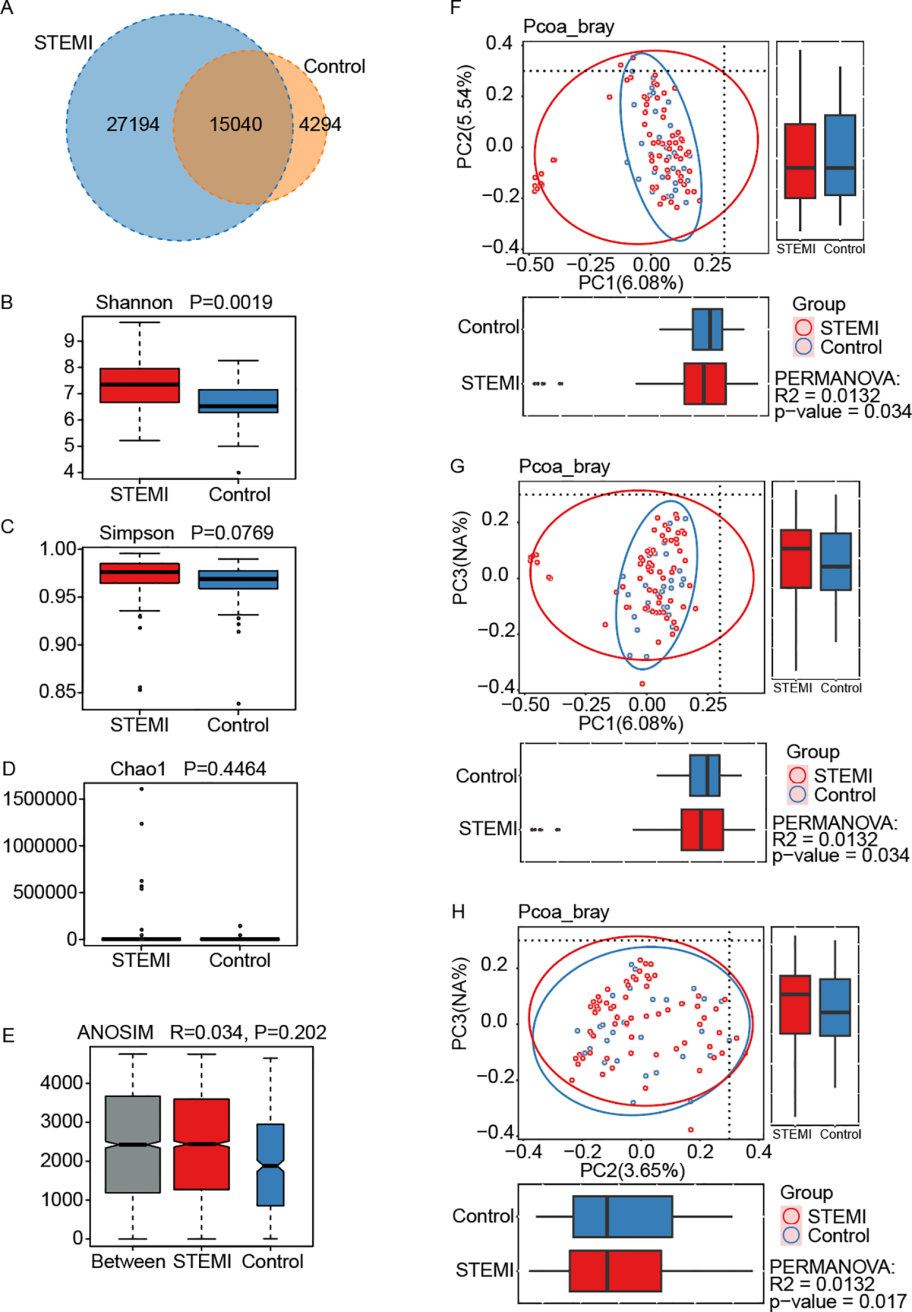
Gut microbiota diversity was assessed in a male population without coronary heart disease (Control, n=32) and in male patients diagnosed with ST-segment elevation myocardial infarction (STEMI, n=66). **(A)** A total of 15,040 OTUs were common to both groups, while 27,194 OTUs were unique to the STEMI group and 4,294 OTUs were specific to the control group. Alpha diversity, reflecting microbial richness and evenness, was evaluated using the Shannon **(B)**, Simpson **(C)**, and Chao1 **(D)** indices, with comparisons between groups performed via the Wilcoxon Rank Sum Test. The resulting P-values were 0.0019 for Shannon, 0.0769 for Simpson, and 0.4464 for Chao1. **(E)** Analysis of Similarity (ANOSIM) demonstrated that intergroup differences exceeded intragroup variations, indicating that the grouping was meaningful. **(F-H)** Beta diversity between the control and STEMI groups was assessed using Principal Component Analysis based on Bray-Curtis distance, revealing significant differences between PC1 and PC2 (p=0.034) **(F)**, PC1 and PC3 (p=0.034) **(G)**, as well as PC2 and PC3 (p=0.017) **(H)**.

In the Venn diagram, a total of 15,040 OTUs were shared between the control and STEMI groups, with the STEMI group harboring a greater number of unique OTUs (27,194) compared to the control group (4,294) (**Figure 2A**). The alpha diversity of the stool microbiome, reflecting the number of species within each sample, varied significantly between the two groups (P = 0.0019 for the Shannon index, P = 0.0769 for the Simpson index, and P = 0.4464 for the Chao1 index) (**Figures 2B-D**). Regarding β-diversity, intergroup differences were more distinct than intragroup variations (R = 0.034, P = 0.202) (**Figure 2E**). Principal component analysis (PCoA) based on Bray-Curtis distance further revealed significant differences in gut microbiota composition between the control and STEMI groups (P = 0.034 for PC1 *vs*. PC2, P = 0.034 for PC1 *vs*. PC3, and P = 0.017 for PC2 *vs*. PC3) (**Figures 2F-H**).

**Figure 3:**
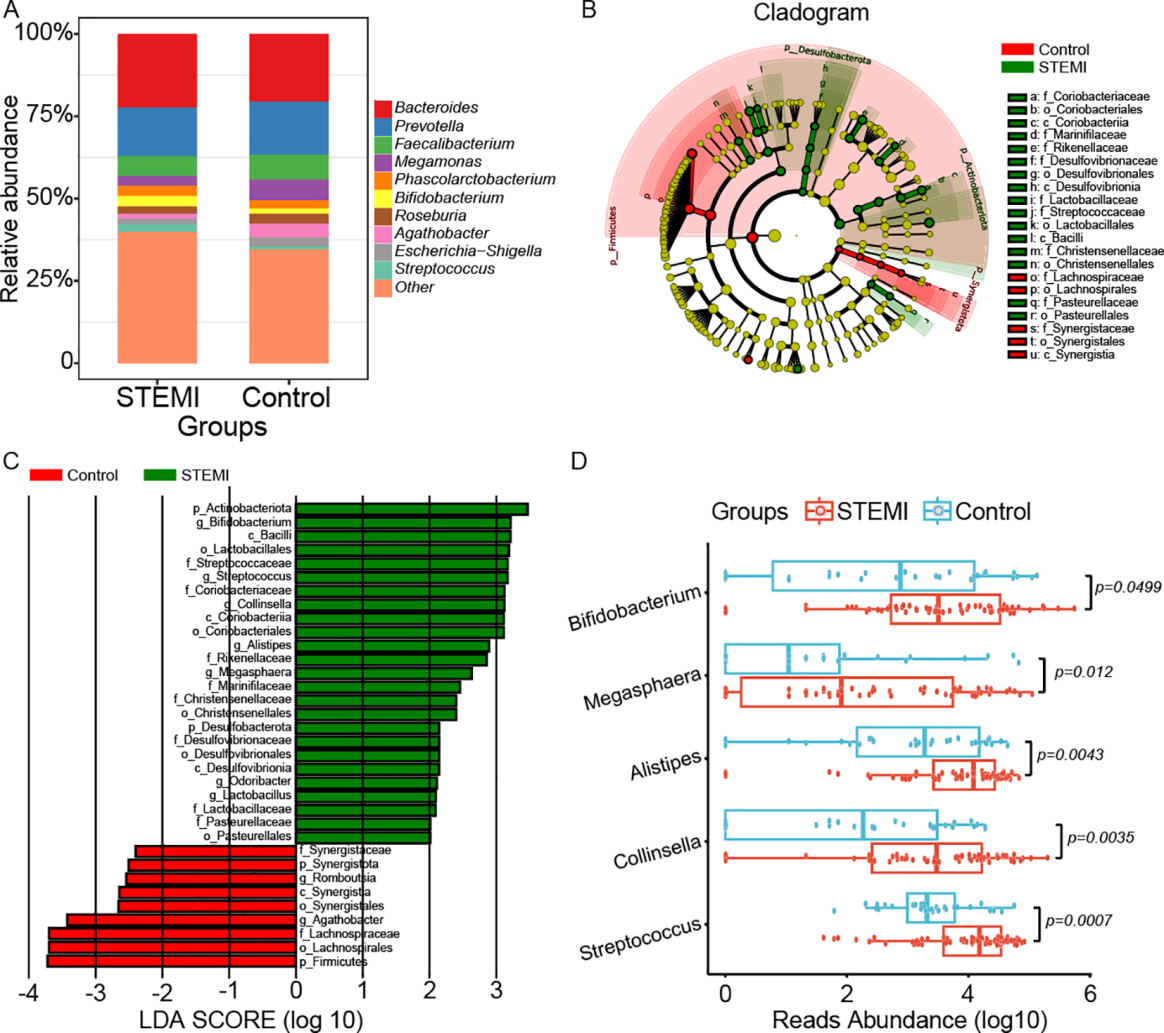
Comparison of gut microbiota composition and key marker genera between the control and STEMI groups. **(A)** Relative abundance histograms depict the distribution of bacterial genera in both groups. The ten most prevalent shared genera, represented by distinct colors, include *Bacteroides, Prevotella, Faecalibacterium, Megamonas, d_Bacteria; p_Firmicutes; c_Clostridia; o_Lachnospirales; f_Lachnospiraceae; g_unidentified, Phascolarctobacterium, Bifidobacterium, Roseburia, Agathobacter*, and *Escherichia-Shigella*, while less abundant genera were collectively classified as “other.” **(B)** A phylogenetic tree was constructed to visualize the hierarchical taxonomic relationships from the phylum to species level for both groups. **(C)** The Wilcoxon rank sum test combined with Linear Discriminant Analysis Effect Size (LEfSe) (P<0.05, LDA>2) identified distinguishing marker genera between the two groups. *Romboutsia* and *Agathobacter* were characteristic of the control group, whereas *Bifidobacterium, Streptococcus, Collinsella, Alistipes, Megasphaera, Odoribacter*, and *Lactobacillus* were significantly enriched in the STEMI group. **(D)** Univariate analysis revealed significant differences in the relative abundance of several genera between the two groups, including *Bifidobacterium* (p=0.0499), *Megasphaera* (p=0.012), *Alistipes* (p=0.0043), *Collinsella* (p=0.0035), and *Streptococcus* (p=0.0007).

Given the limitations of 16S rDNA amplicon pyrosequencing, our analysis primarily focused on genus-level microbial composition. **Figure 3A** and **Supplementary Figure 1** illustrated the distribution of gut microbiota at this taxonomic level. Both groups exhibited a predominance of *Bacteroides, Prevotella, Faecalibacterium, Megamonas, Phascolarctobacterium, Bifidobacterium, Roseburia, Agathobacter, Escherichia−Shigella*, and *Streptococcus*. However, *Bacteroides*, *Phascolarctobacterium*, *Bifidobacterium*, and *Streptococcus* were more enriched in the STEMI group, whereas *Prevotella, Faecalibacterium, Megamonas, Roseburia, Agathobacter*, and *Escherichia−Shigella* were relatively more abundant in the control group. The Wilcoxon rank sum test (LEfSe) (P < 0.05, LDA > 2) identified key genera distinguishing the two groups, with the STEMI group exhibiting a greater number of biomarker bacteria than the control group (**Figures 3B, C**). Specifically, *Bifidobacterium, Streptococcus, Collinsella, Alistipes, Megasphaera, Odoribacter*, and *Lactobacillus* were characteristic genera of the STEMI group, whereas *Romboutsia* and *Agathobacter* were indicative of the control group (**Figure 3C**). Additionally, statistical significance was confirmed for five differentially abundant genera, with P values of 0.0499, 0.012, 0.0043, 0.0035, and 0.0007 for *Bifidobacterium, Megasphaera, Alistipes, Collinsella*, and *Streptococcus*, respectively (**Figure 3D**).

To further explore functional differences between the two groups, we performed Picrust2 analysis, which predicted variations in KEGG, METACYC, CAZY, and GMM modules. The top 10 most significantly altered pathways were presented in **Supplementary Figures 2A-D**. Notably, several KEGG pathways associated with metabolic regulation were disrupted in the STEMI group, including the insulin signaling pathway (P = 0.0006), thiamine metabolism (P = 0.0017), terpenoid backbone biosynthesis (P = 0.0039), and porphyrin and chlorophyll metabolism (P = 0.0056) (**Supplementary Figure 2A**). Specifically, all these metabolic pathways were computational and should be interpreted as hypothesis-generating rather than confirmatory.

### 3.3 Differences in diversity, composition, and metabolic pathways of gut microbiota between the Non-MACEs group and the MACEs group

**Figure 4:**
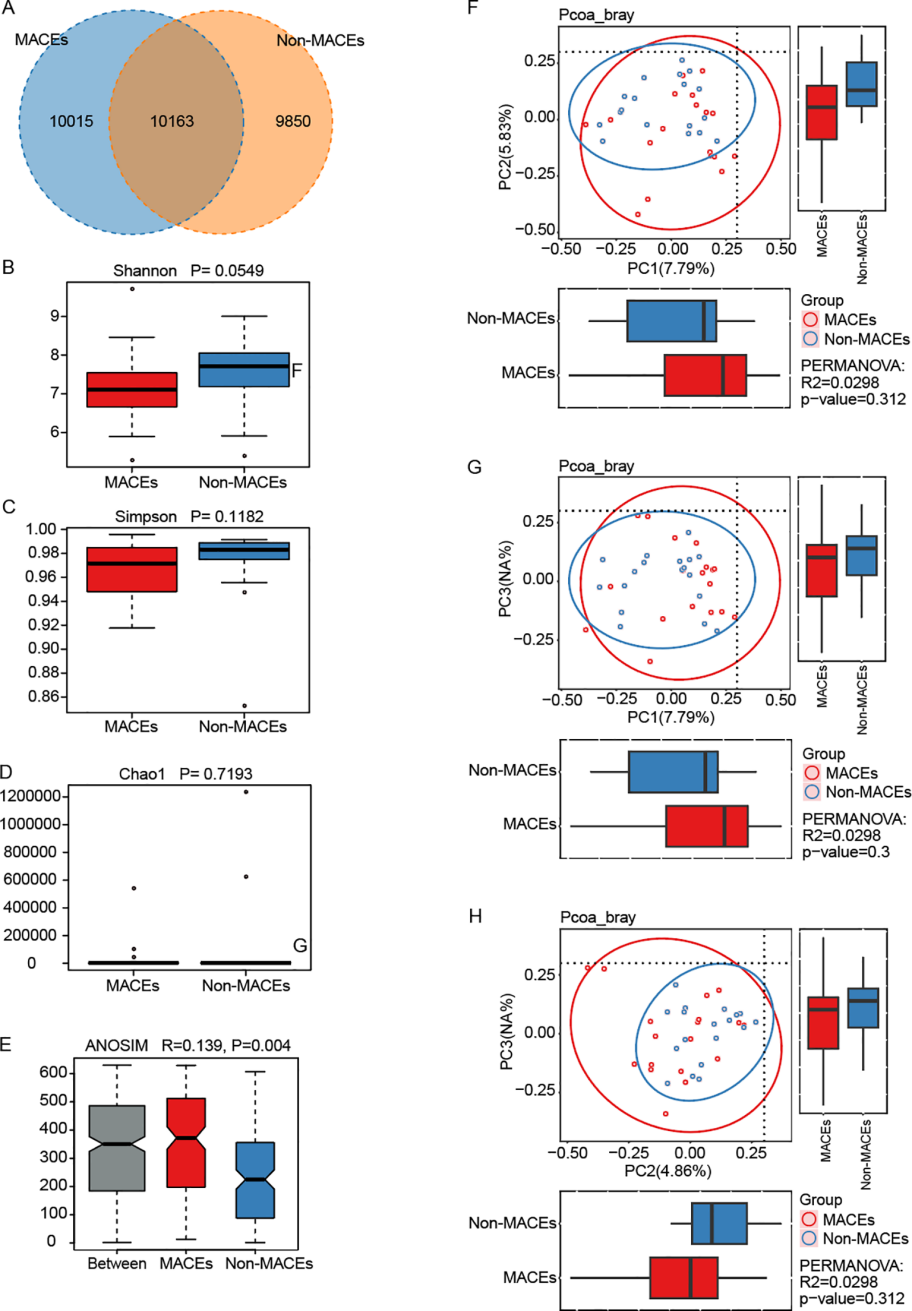
Gut microbiota diversity in patients with STEMI with and without major adverse cardiovascular events (MACEs). The study compared patients who experienced MACEs (n=18) with age-matched STEMI patients who did not (Non-MACEs, n=18). **(A)** A total of 10,163 operational taxonomic units (OTUs) were shared between the two groups, with 10,015 OTUs uniquely identified in the MACEs group and 9,850 in the Non-MACEs group. **(B-D)** Alpha diversity, reflecting microbial richness and evenness, was assessed using the Shannon, Simpson, and Chao1 indices, calculated via the Wilcoxon Rank Sum Test. The corresponding P-values were 0.0549 for Shannon, 0.1182 for Simpson, and 0.7193 for Chao1. **(E)** Analysis of Similarity (ANOSIM) confirmed that intergroup differences exceeded intragroup variations, supporting the validity of the group classification. **(F-H)** Beta diversity was evaluated through Principal Component Analysis (PCA) based on Bray-Curtis distance. However, no statistically significant differences were observed in comparisons between PC1 and PC2 (p=0.312) **(F)**, PC1 and PC3 (p=0.300) **(G)**, or PC2 and PC3 (p=0.312) **(H)**.

In the Venn diagram, 10,163 OTUs were shared between the Non-MACEs and MACEs groups, with the MACEs group possessing a greater number of unique OTUs (10,015) in comparison to the Non-MACEs group (9,850) (**Figure 4A**). The alpha diversity of the gut microbiome, reflecting the species richness within each sample, showed no statistically significant differences between the two groups (P = 0.0549 for the Shannon index, P = 0.1182 for the Simpson index, and P = 0.7193 for the Chao1 index) (**Figures 4B-D**). In terms of β-diversity, intergroup differences were more pronounced than intragroup variations (R = 0.139, P = 0.004) (**Figure 4E**). However, Principal Component Analysis (PCoA) based on Bray-Curtis distance did not reveal significant compositional differences in gut microbiota between the Non-MACEs and MACEs groups (P = 0.312 for PC1 *vs*. PC2, P = 0.300 for PC1 *vs*. PC3, and P = 0.312 for PC2 *vs*. PC3) (**Figures 4F-H**).

**Figure 5:**
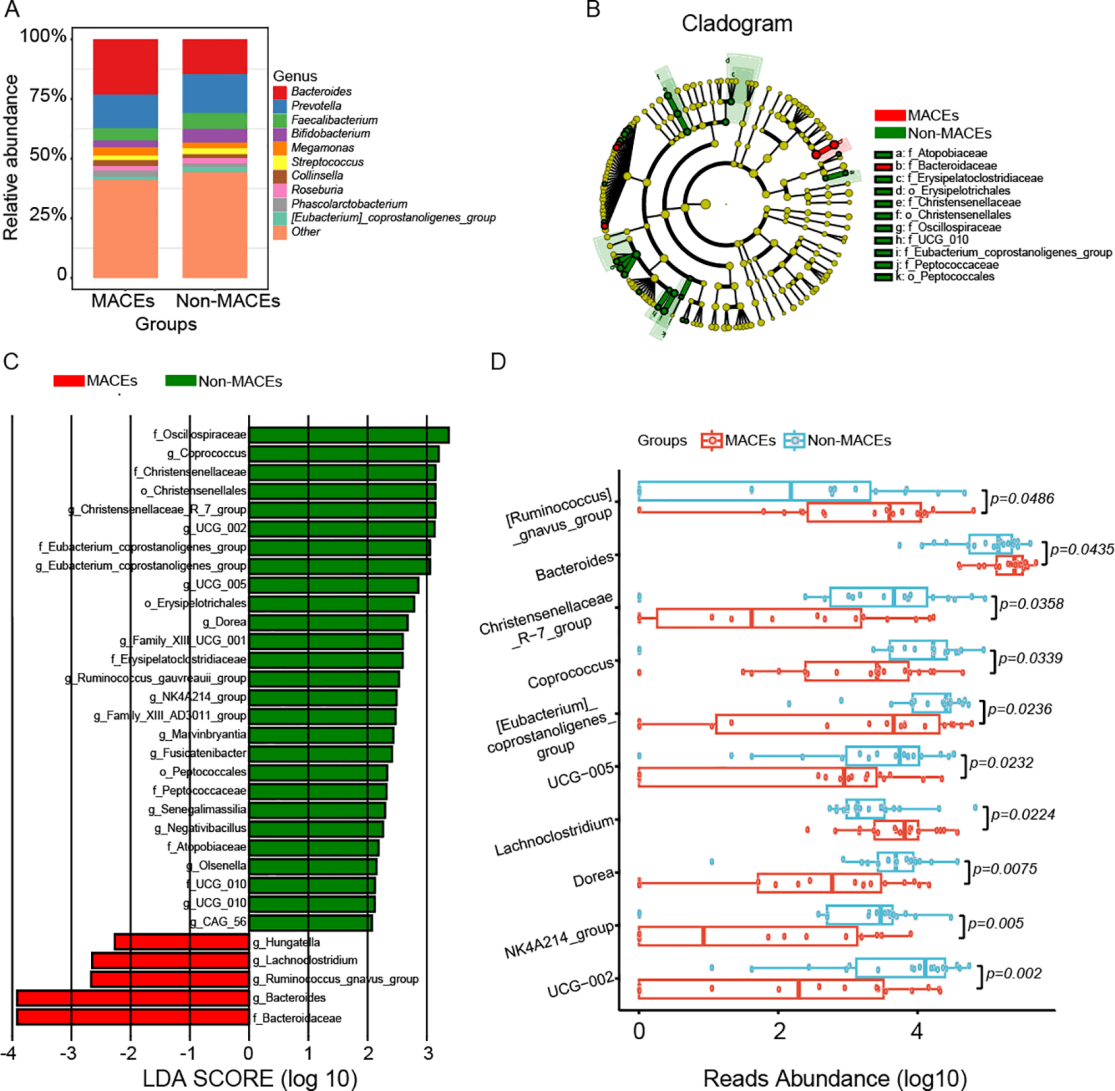
Composition and key marker genera of gut microbiota in the Non-MACEs and MACEs groups. **(A)** The relative abundance histograms illustrated the distribution of all detected genera across both groups. The 10 most prevalent shared genera, depicted in distinct colors, include *Bacteroides*, *Prevotella*, *Faecalibacterium*, *Bifidobacterium*, *Megamonas*, *Streptococcus*, *Collinsella*, *Roseburia*, *Phascolarctobacterium*, and *[Eubacterium]_coprostanoligenes_group*, whereas genera with lower relative abundance are categorized as ‘other.’ **(B)** The phylogenetic tree visualizes the hierarchical classification of marker taxa from the phylum to species level within the two groups. **(C)** The Wilcoxon rank sum test (LEfSe) (P<0.05, LDA>2) identified key marker genera distinguishing the Non-MACEs and MACEs groups. In the Non-MACEs group, significant marker genera included *Coprococcus*, *Christensenellaceae_R_7_group*, *UCG_002*, *Eubacterium_coprostanoligenes_group*, *UCG_005*, *Dorea*, *Family_XIII_UCG_001*, *Ruminococcus_gauvreauii_group*, *NK4A214_group*, *Family_XIII_AD3011_group*, *Marvinbryantia*, *Fusicatenibacter*, *Senegalimassilia*, *Negativibacillus*, *Olsenella*, *UCG_010*, and *CAG_56*. In contrast, *Hungatella*, *Lachnoclostridium*, *Ruminococcus_gnavus_group*, and *Bacteroides* were identified as markers for the MACEs group. **(D)** The Univariate Test revealed significant differences in several genera between the two groups, including *[Ruminococcus]_gnavus_group* (p=0.0486), *Bacteroides* (p=0.0435), *Christensenellaceae_R-7_group* (p=0.0358), *Coprococcus* (p=0.0339), *[Eubacterium]_coprostanoligenes_group* (p=0.0236), *UCG-005* (p=0.0232), *Lachnoclostridium* (p=0.0224), *Dorea* (p=0.0075), *NK4A214_group* (p=0.005), and *UCG-002* (p=0.002).

Due to the inherent limitations of 16S rDNA amplicon pyrosequencing, our analysis predominantly focused on microbial composition at the genus level. **Figure 5A** and **Supplementary Figure 3** illustrated the taxonomic distribution of gut microbiota at this resolution. Both the Non-MACEs and MACEs groups were characterized by the presence of *Bacteroides, Prevotella, Faecalibacterium, Bifidobacterium, Megamonas, Streptococcus, Collinsella, Roseburia, Phascolarctobacterium*, and *[Eubacterium]_coprostanoligenes_group*. However, *Bacteroides*, *Megamonas*, *Collinsella*, and *Phascolarctobacterium* were more prevalent in the MACEs group, whereas *Prevotella, Faecalibacterium, Bifidobacterium, Streptococcus, Roseburia*, and *[Eubacterium]_coprostanoligenes_group* were found in higher abundance in the Non-MACEs group. The Wilcoxon rank sum test (LEfSe) (P < 0.05, LDA > 2) identified distinct marker genera differentiating the two groups, with the Non-MACEs group exhibiting a greater number of characteristic bacterial taxa compared to the MACEs group (**Figures 5B, C**). Specifically, *Coprococcus, Christensenellaceae_R_7_group, UCG_002, Eubacterium_coprostanoligenes_group, UCG_005, Dorea, Family_XIII_UCG_001, Ruminococcus_gauvreauii_group, NK4A214_group, Family_XIII_AD3011_group, Marvinbryantia, Fusicatenibacter, Senegalimassilia, Negativibacillus, Olsenella, UCG_010*, and *CAG_56* were signature genera for the Non-MACEs group, while *Hungatella, Lachnoclostridium, Ruminococcus_gnavus_group*, and *Bacteroides* were representative of the MACEs group (**Figure 5C**). Moreover, statistical analysis confirmed significant differences in 10 genera, with P values of 0.0486, 0.0435, 0.0358, 0.0339, 0.0236, 0.0232, 0.0224, 0.0075, 0.005, and 0.002 for *[Ruminococcus]_gnavus_group, Bacteroides, Christensenellaceae_R-7_group, Coprococcus, [Eubacterium]_coprostanoligenes_group, UCG-005, Lachnoclostridium, Dorea, NK4A214_group*, and *UCG-002*, respectively (**Figure 5D**).

To further investigate functional discrepancies between the groups, Picrust2 analysis was conducted to predict variations in KEGG, CAZY, METACYC, and GMM functional modules, with 5, 5, 9, and 5 significantly different pathways identified, respectively, as presented in **Supplementary Figure 4A-D**. Notably, metabolic pathways such as chloroalkane and chloroalkene degradation (P = 0.0499), carotenoid biosynthesis (P = 0.0435), and steroid biosynthesis (P = 0.0046) were implicated in organic compound metabolism, while the mRNA surveillance pathway (P = 0.0142) was associated with signal transduction and regulatory processes (**Supplementary Figure 4A**). It is important to note that all of these metabolic pathways were inferred through computational analysis and should be regarded as exploratory hypotheses rather than definitive findings.

### 3.4 Blood lipid profile in the non-MACEs group and the MACEs group

**Figure 6:**
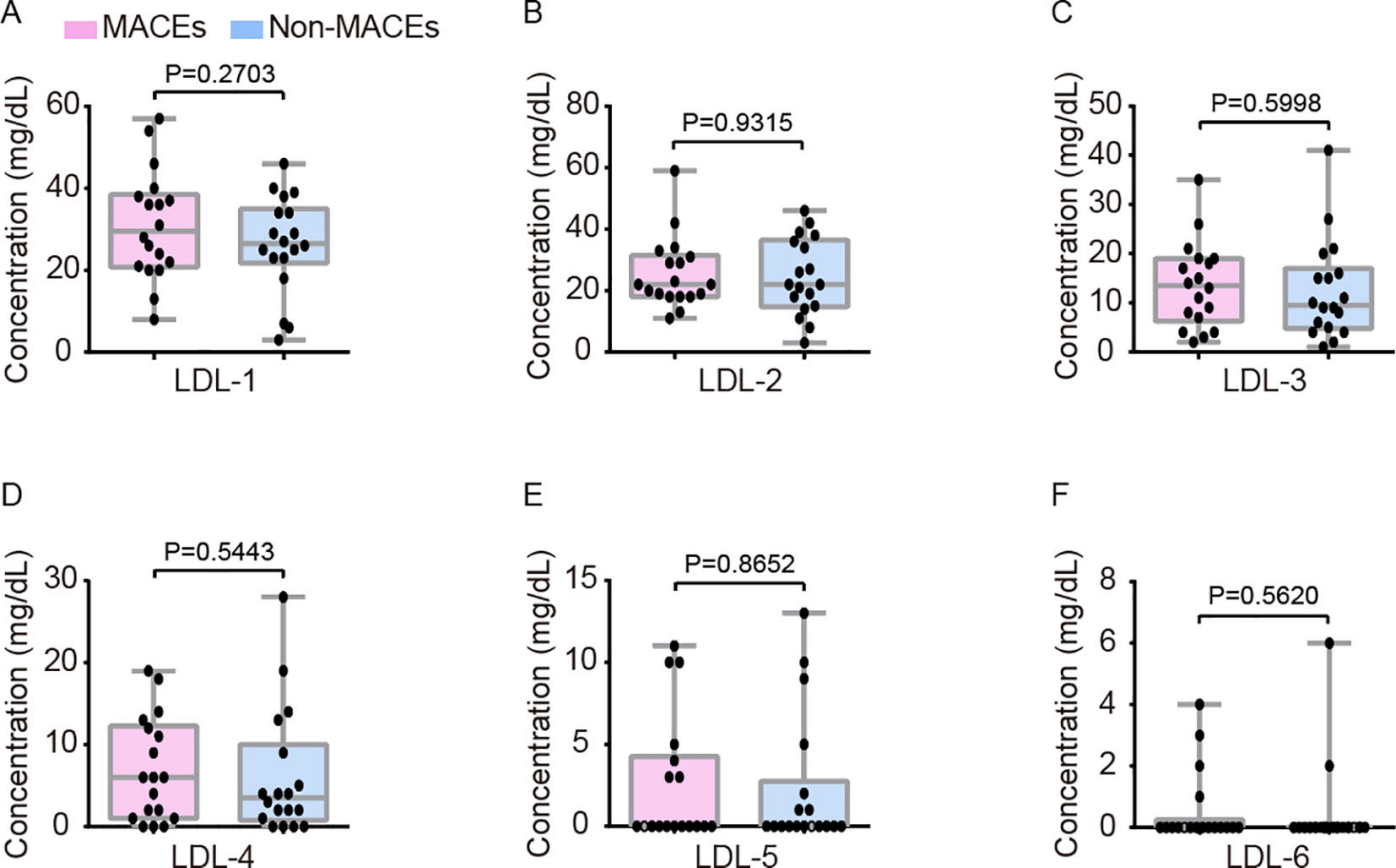
Comparative assessment of LDL-C subfraction levels between the Non-MACEs and MACEs groups, covering LDLC-1 **(A)**, LDLC-2 **(B)**, LDLC-3 **(C)**, LDLC-4 **(D)**, LDLC-5 **(E)**, and LDLC-6 **(F)**. The analysis revealed no statistically significant differences in LDL-C subfractions between the two groups. LDL-C, low-density lipoprotein cholesterol.

**Table 2** presented the blood lipid profiles of patients with STEMI, stratified by the presence or absence of MACEs. No statistically significant differences were observed in TC, TG, HDL-C, and LDL-C levels between the MACEs and Non-MACEs groups. Furthermore, none of the LDL-C subfractions exhibited significant variation between the two groups (**Figure 6**).

### 3.5 Correlations analysis among the top 10 marker genera and six LDL-C subfractions in patients with STEMI with MACEs

**Figure 7:**
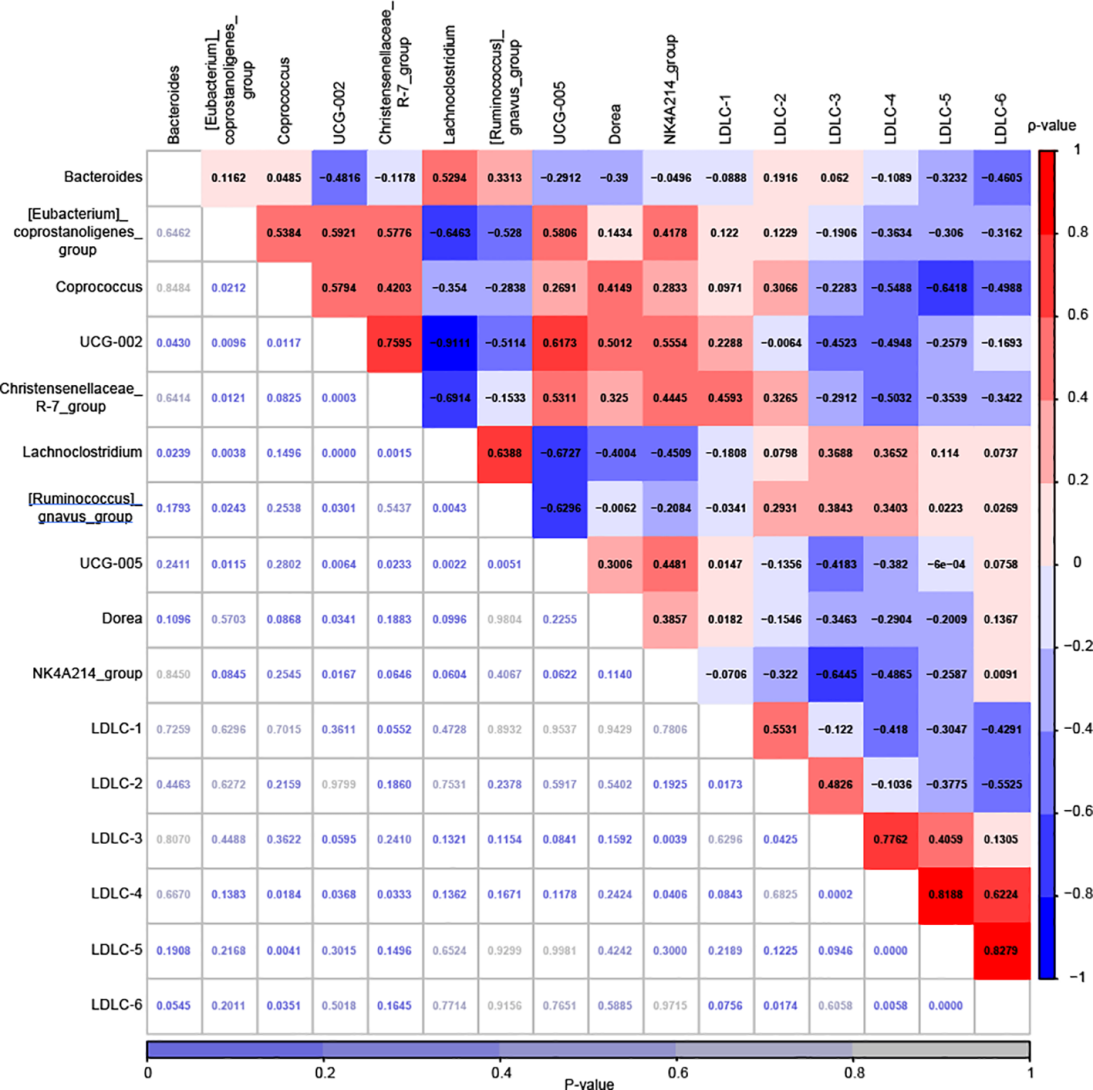
Correlation analysis was conducted to examine the relationship between the relative abundances of 10 marker genera and the plasma levels of six LDL-C subfractions in the MACEs group, using Spearman’s Rank Correlation. The correlation coefficients are displayed in various colors, with the corresponding scale shown on the right. The P-values, which are not highlighted, are listed at the bottom along with their scale.

As illustrated in **Figure 7**, both significant positive and negative correlations were detected among the top 10 marker genera. Notable correlations included a negative association between *Bacteroides* and *UCG-002* (ρ=-0.4816, P<0.05), as well as a positive correlation between *Bacteroides* and *Lachnoclostridium* (ρ=0.5294, P<0.05). Additionally, *[Eubacterium]_coprostanoligenes_group* demonstrated a positive correlation with *Coprococcus* (ρ=0.5384, P<0.05), *UCG-002* (ρ=0.5921, P<0.01), *Christensenellaceae_R-7_group* (ρ=0.5776, P<0.05), and *UCG-005* (ρ=0.5806, P<0.05), while exhibiting a negative association with *Lachnoclostridium* (ρ=-0.6463, P<0.01) and *[Ruminococcus]_gnavus_group* (ρ=-0.5280, P<0.05). Furthermore, *Coprococcus* displayed a positive correlation with *UCG-002* (ρ=0.5794, P<0.05), whereas *UCG-002* showed significant positive associations with *Christensenellaceae_R-7_group* (ρ=0.7595, P<0.001), *UCG-005* (ρ=0.6173, P<0.01), *Dorea* (ρ=0.5012, P<0.01), and *NK4A214_group* (ρ=0.5554, P<0.05), but was negatively correlated with *Lachnoclostridium* (ρ=-0.9111, P<0.001) and *[Ruminococcus]_gnavus_group* (ρ=-0.5114, P<0.05). Similarly, *Christensenellaceae_R-7_group* exhibited a negative correlation with *Lachnoclostridium* (ρ=-0.6914, P<0.01) and a positive correlation with *UCG-005* (ρ=0.5311, P<0.05). Additional significant associations included a positive correlation between *Lachnoclostridium* and *[Ruminococcus]_gnavus_group* (ρ=0.6388, P<0.01) and a negative correlation with *UCG-005* (ρ=-0.6727, P<0.01), while *[Ruminococcus]_gnavus_group* also displayed a negative correlation with *UCG-005* (ρ=-0.6296, P<0.01). Moreover, we further examined the relationship between gut microbiota and LDL-C subfractions. Interestingly, significant negative correlations were identified between *Coprococcus* and LDLC-4 (ρ=-0.5488, P<0.05), LDLC-5 (ρ=-0.6418, P<0.01), and LDLC-6 (ρ=-0.4988, P<0.05). Similarly, *UCG-002* (ρ=-0.4948, P<0.05) and *Christensenellaceae_R-7_group* (ρ=-0.5032, P<0.05) were negatively associated with LDLC-4. Notably, no significant positive correlations were observed (**Figure 7**).

### 3.6 Correlations analysis among the top 10 marker genera and six LDL-C subfractions in patients with STEMI without MACEs

**Figure 8:**
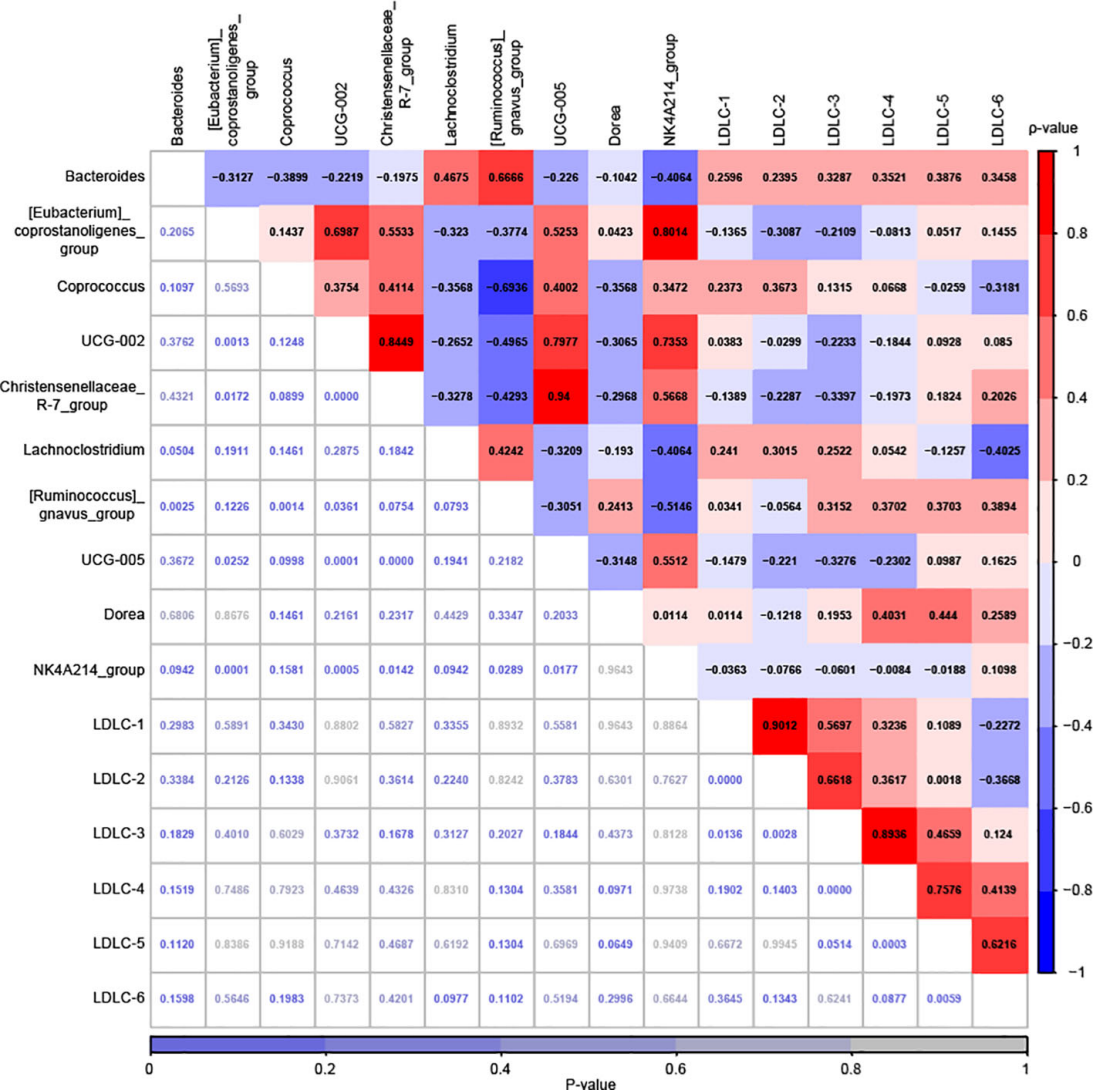
Correlation analysis among the relative abundances of 10 marker genera and the plasma levels of 6 LDL-C subfraction levels for the Non-MACEs groups, which was performed by Spearman’s Rank Correlation Analysis. The correlation coefficients are highlighted in various colors, with the corresponding scale shown on the right. The P-values, which are not highlighted, are listed at the bottom along with their scale.

As shown in **Figure 8**, both significantly positive and negative correlations were observed among the top 10 marker genera, including between *Bacteroides* and *[Ruminococcus]_gnavus_group* (ρ=0.6666, *P*<0.01), between *[Eubacterium]_coprostanoligenes_group* and *UCG-002* (ρ=0.6987, *P*<0.01), between *[Eubacterium]_coprostanoligenes_group* and *Christensenellaceae_R-7_group* (ρ=0.5533, *P*<0.05), between *[Eubacterium]_coprostanoligenes_group* and *UCG-005* (ρ=0.5253, *P*<0.05), between *[Eubacterium]_coprostanoligenes_group* and *NK4A214_group* (ρ=0.8014, *P*<0.001), between *Coprococcus* and *[Ruminococcus]_gnavus_group* (ρ=-0.6936, *P*<0.01), between *UCG-002* and *Christensenellaceae_R-7_group* (ρ=0.8449, *P*<0.001), between *UCG-002* and *[Ruminococcus]_gnavus_group* (ρ=-0.4965, *P*<0.05), between *UCG-002* and *UCG-005* (ρ=0.7977, *P*<0.001), between *UCG-002* and *NK4A214_group* (ρ=0.7353, *P*<0.001), between *Christensenellaceae_R-7_group* and *UCG-005* (ρ=0.940, *P*<0.001), between *Christensenellaceae_R-7_group* and *NK4A214_group* (ρ=0.5668, *P*<0.05), between *[Ruminococcus]_gnavus_group* and *NK4A214_group* (ρ=-0.5146, *P*<0.05), and between *UCG-005* and *NK4A214_group* (ρ=0.5512, *P*<0.05). However, we did not observe any significant correlations between gut microbiota and LDL-C subfractions, which was inconsistent with the MACEs group (**Figure 8**).

### 3.7 Predictive value of the top 10 marker genera and six LDL-C subfractions

The ROC curve analysis demonstrated the predictive potential of various gut microbiota genera, with AUC values as follows: *Bacteroides* (AUC = 0.698, 95% CI: 0.524-0.871), *[Eubacterium]_coprostanoligenes_group* (AUC = 0.722, 95% CI: 0.548-0.897), *Coprococcus* (AUC = 0.727, 95% CI: 0.554-0.900), *UCG-002* (AUC = 0.759, 95% CI: 0.595-0.924), *Christensenellaceae_R-7_group* (AUC = 0.752, 95% CI: 0.593-0.910), *Lachnoclostridium* (AUC = 0.722, 95% CI: 0.545-0.900), *[Ruminococcus]_gnavus_group* (AUC = 0.677, 95% CI: 0.498-0.857), *UCG-005* (AUC = 0.685, 95% CI: 0.503-0.868), *Dorea* (AUC = 0.750, 95% CI: 0.581-0.919), and *NK4A214_group* (AUC = 0.779, 95% CI: 0.625-0.934) (**Table 3**). Among these key marker genera, *UCG-002*, *Christensenellaceae_R-7_group*, and *NK4A214_group* exhibited relatively superior predictive performance, with AUC values exceeding 0.75. Additionally, the predictive capacities of six LDL-C subfractions were evaluated: LDLC-1 (AUC = 0.571, 95% CI: 0.380–0.762), LDLC-2 (AUC = 0.509, 95% CI: 0.315–0.704), LDLC-3 (AUC = 0.552, 95% CI: 0.361–0.744), LDLC-4 (AUC = 0.560, 95% CI: 0.368–0.753), LDLC-5 (AUC = 0.515, 95% CI: 0.324–0.707), and LDLC-6 (AUC = 0.551, 95% CI: 0.360–0.741) (**Table 3**). Notably, in comparison to gut microbiota markers, LDL-C subfractions exhibited lower predictive powers for MACEs.

**Table 3:** Predictive values of gut microbiota and LDL-C subfractions for MACEs.

Variables	AUC (95% CI)	Cutoff value	Sensitivity (%)	Specificity (%)
Gut microbiota				
Bacteroides	0.698	3.382794	0.556	0.833
[Eubacterium]_coprostanoligenes_group	0.722	1.858472	0.667	0.833
Coprococcus	0.727	1.552907	0.667	0.778
UCG-002	0.759	1.916167	0.833	0.667
Christensenellaceae_R-7_group	0.752	0.190762	0.556	0.833
Lachnoclostridium	0.722	1.662418	0.667	0.833
[Ruminococcus]_gnavus_group	0.677	0.03878	0.889	0.5
UCG-005	0.685	1.641221	0.889	0.556
Dorea	0.75	1.249311	0.667	0.833
NK4A214_group	0.779	0.483268	0.667	0.833
Low-density lipoprotein cholesterol subtyping				
LDLC-1	0.571	35	0.444	0.778
LDLC-2	0.509	16.5	0.889	0.278
LDLC-3	0.552	12	0.556	0.611
LDLC-4	0.56	5.5	0.556	0.722
LDLC-5	0.515	2.5	0.389	0.778
LDLC-6	0.551	0.5	0.222	0.889

## 4 Discussion

This study investigated the differences in gut microbiota profiles and the distribution of LDL-C subfractions between the Non-MACEs group and the MACEs group, and elucidated the interplay between gut microbiota and LDL-C subfractions in these two groups. The primary findings were as follows: (1) there were significant differences in gut microbiota composition but not gut microbiota diversity between the Non-MACEs group and the MACEs group, with the Non-MACEs group featuring a greater number of marker bacteria compared to the MACEs group; (2) there were no significant differences in TC, TG, HDL-C, LDL-C, LDLC-1, LDLC-2, LDLC-3, LDLC-4, LDLC-5, and LDLC-6 between the Non-MACEs group and the MACEs group; (3) multiple significantly negative correlations were identified between gut microbiota and LDL-C subfractions in the MACEs group, while none of significant correlations were observed in the Non-MACEs group; (4) the predictive performances of the top 10 marker genera were much better than the six LDL-C subfractions, and the AUC values of *UCG-002*, *Christensenellaceae_R-7_group*, and *NK4A214_group* were greater than 0.75. These findings suggested a potential role of gut microbiota in post-STEMI prognosis, emphasizing its relevance in cardiovascular risk stratification.

Statistical analyses revealed significant differences in the abundance of ten genera, with P values indicating distinct microbiota alterations between the Non-MACEs group and the MACEs group. Notably, [*Ruminococcus]_gnavus_group* and *Bacteroides*, both identified as MACEs-associated genera, demonstrated significant associations (P = 0.0486 and P = 0.0435, respectively), consistent with previous research linking these taxa to systemic inflammation and atherogenic progression (42–44). The microbial shifts observed in the MACEs group suggest potential pathogenic mechanisms contributing to adverse cardiovascular outcomes. *Ruminococcus_gnavus_group* had been linked to increased TMAO production, a metabolite derived from dietary choline and carnitine, which has been shown to enhance platelet activation, leading to thrombogenesis and heightened cardiovascular risk (45–48). *Bacteroides*, another key genus enriched in the MACEs group, plays a role in bile acid deconjugation, potentially exacerbating lipid dysregulation and systemic inflammation, thereby further predisposing patients to recurrent ischemic events (49–53). The observed microbial composition in the MACEs group suggests an enhanced inflammatory and pro-thrombotic state, reinforcing the hypothesis that gut microbiota may serve as a modifiable risk factor in post-STEMI prognosis. However, the proposed involvement of TMAO and bile acid pathways was inferred from the existing literature linking *Ruminococcus_gnavus_group* and *Bacteroides*. Future studies should focus on elucidating the mechanistic pathways linking these bacterial taxa to cardiovascular pathology by incorporating targeted metabolomics (e.g., LC-MS/MS analysis of TMAO and deoxycholic acid). Moreover, it was also interesting to explore the potential microbiome-targeted interventions, such as probiotics, dietary modifications, or pharmacological strategies aimed at reducing TMAO levels and systemic inflammation (54–56).

In patients with STEMI with MACEs, strong correlations were observed among several key genera. Notably, *Bacteroides* was negatively correlated with *UCG-002*, while positively correlated with *Lachnoclostridium*. Given that *Bacteroides* is involved in bile acid metabolism and short-chain fatty acid (SCFA) production (57–59), its negative association with *UCG-002*, a genus previously linked to anti-inflammatory effects (60), may suggest an altered inflammatory milieu favoring cardiovascular events. Meanwhile, *Lachnoclostridium* showed a negative correlation with *[Eubacterium]_coprostanoligenes_group*, *UCG-002*, and *Christensenellaceae_R-7_group*, but a positive association with *[Ruminococcus]_gnavus_group*. These findings aligned with prior studies that implicated *Lachnoclostridium* in increased intestinal permeability and pro-atherogenic lipid profiles, potentially exacerbating the risk of MACEs (61). In contrast, the non-MACEs group displayed a different pattern of microbial interactions. For example, *[Eubacterium]_coprostanoligenes_group* was positively correlated with *UCG-002*, *Christensenellaceae_R-7_group*, *UCG-005*, and *NK4A214_group*, indicating a cooperative role in maintaining gut homeostasis and potentially exerting cardioprotective effects through SCFA production and immune modulation (44). The robust positive association between *Christensenellaceae_R-7_group* and *UCG-005* (ρ = 0.940, P < 0.001) suggested a tightly linked network contributing to intestinal barrier integrity and reduced systemic inflammation, factors that might protect against adverse cardiovascular outcomes (62, 63). Furthermore, *Ruminococcaceae_UCG-005* and *Christensenellaceae R-7 group* have been positively associated with healthy gut profiles, such as enhanced SCFA production, reduced triglycerides, and lower fecal bile acid concentrations, in both adults and pediatric populations (64–66).

The interplay between gut microbiota and lipid metabolism was further explored through the correlation analysis between gut microbiota and LDL-C subfractions. In patients with STEMI with MACE, we observed significantly negative correlations between *Coprococcus* and LDLC-4 (ρ=-0.5488, P<0.05), between *Coprococcus* and LDLC-5 (ρ=-0.6418, P<0.01), between *Coprococcus* and LDLC-6 (ρ=-0.4988, P<0.05), between *UCG-002* and LDLC-4 (ρ=-0.4948, P<0.05), and between *Christensenellaceae_R-7_group* and LDLC-4 (ρ=-0.5032, P<0.05). These findings suggested that certain gut microbial genera might play a protective role in modulating the distribution of LDL-C subfractions, particularly the more atherogenic small, dense LDL particles. Interestingly, *Coprococcus*, *Christensenellaceae_R-7_group*, and *UCG-002* have been previously implicated in SCFA production, which has beneficial effects on lipid metabolism and endothelial function (67–70). Notably, SCFAs not only serve as substrates in lipid metabolism but also function as regulatory factors in the modulation of lipid metabolism. Li et al. demonstrated that butyric acid enhances fatty acid oxidation in brown adipose tissue, thereby alleviating diet-induced obesity and insulin resistance (71). Additionally, the migration and recruitment of immune cells to endothelial cells has also been influenced by SCFAs, a critical step in the pathogenesis of inflammatory diseases such as atherosclerosis (72). Several studies have shown that these effects are mediated through the regulation of adhesion molecule expression on immune and endothelial cells via activating FFA2 and FFA3 receptors (73–75). Collectively, these negative correlations observed in our study might reflect a potential role of these microbes in limiting the accumulation of highly atherogenic LDL-C subfractions through SCFA-mediated pathways. However, due to the limitation of research funds, the serum levels of SCFAs were lacking in this study, which significantly undermined the reliability of our hypothesis. Our findings should be validated in future studies incorporating multi-omics approaches (e.g., metabolomics and host transcriptomics) and experimental models to better identify the causal pathways linking gut microbiota to MACEs.

The findings of this study highlighted the potential of gut microbiota as a novel biomarker for cardiovascular risk stratification. The superior predictive performance of *UCG-002*, *Christensenellaceae_R-7_group*, and *NK4A214_group* compared to LDL-C subfractions suggested that microbial signatures might provide valuable complementary information beyond traditional lipid metrics. This was particularly relevant given the growing recognition of the gut-heart axis and its implications for cardiovascular disease prevention and management (6, 76, 77). However, several limitations of this study should be acknowledged. Firstly, the involvement of TMAO, bile acids, and SCFAs was hypothesis-generating, and they were inferred from the existing literature linking specific gut microbiota (e.g., *Ruminococcus_gnavus_group*, *Bacteroides, and Christensenellaceae_R-7_group*). We should incorporate targeted metabolomics (e.g., LC-MS/MS analysis) to validate these mechanistic hypotheses between patients with STEMI with and without MACEs. Secondly, an absence of mechanistic studies between gut microbiota and lipid metabolism in the context of cardiovascular risk. Furthermore, we should explore how these bacteria and their metabolites regulate LDL-C subfractions dynamics. Moreover, although steroid biosynthesis was predicted as a significantly different metabolic pathways between the MACEs group and the Non-MACEs group by the Picrust2 analysis, and exogenous steroids (e.g. anabolic or corticosteroids) were associated with higher cardiovascular event risk such as MI, heart failure, and arrhythmias (78, 79), multi-omics approaches (e.g., metagenomics, metabolomics, and host transcriptomics) and experimental models should also be incorporated to better dissect the causal pathways linking gut microbiota to lipid metabolism and systemic inflammation in patients with STEMI with MACEs. Thirdly, although we restricted the population to local residents and rice as a staple food, other potential confounding factors such as lifestyle and genetic predispositions were not fully accounted for, which may influence gut microbial composition and lipid metabolism. Fourthly, given that the study cohort consisted exclusively of local residents with rice as a staple food, our findings may reflect gut microbiota and lipid metabolism profiles specific to the high-carbohydrate pattern. Prior studies have shown that such diets can selectively promote the growth of SCFA-producing taxa. For instance, Coprococcus species, a SCFA-producing genus, has been observed to increase in plant-based dietary interventions and is linked to improved metabolic outcomes (80). However, these associations may differ in Western populations with higher fat and protein intake. Thus, while our results offer mechanistic insights into post-STEMI prognosis in a specific dietary setting, further multicenter studies across diverse nutritional backgrounds are warranted to verify their broader applicability. Lastly, the relatively small sample size necessitates further validation in larger cohorts with diverse populations.

In conclusion, our study suggests that gut microbiota, particularly *UCG-002*, *Christensenellaceae_R-7_group*, and *NK4A214_group*, exhibit superior predictive performance for MACEs compared to LDL-C subfractions. Our findings underscored the potential of gut microbial biomarkers in cardiovascular risk assessment and paved the way for future microbiota-targeted interventions in cardiovascular disease management.

## Data Availability

The raw reads of 16S rRNA sequencing are available NCBI at accession number PRJNA1230799. Other datasets generated during the current study are available from the corresponding authors upon reasonable request.
