# Supplementary material for: Prognostic value of gut microbiota and low-density lipoprotein cholesterol subfractions in patients with ST-segment elevation myocardial infarction
Source: Front Immunol. 2025 Aug 13;16:1610001. doi: 10.3389/fimmu.2025.1610001 (PMC12381562; doi:10.3389/fimmu.2025.1610001)
Supplement: Supplementary file 1 [file DataSheet1.pdf]

Prognostic value of gut microbiota and low-density lipoprotein cholesterol subfractions in patients with ST-segment elevation myocardial infarction

Siliang Xia[1†], Yun Liu[1†], Mengzhu Wang[2†], Dandan Liu[1], Xiaobing Zhang[1], Ling Lin[2], Ming Wen[2], Shushen Ji[2], Jiaying Li[3*], Xiangming Zhang[1*] and Huihui Jiang[2*]

[1]Department of Cardiology, Nanjing Jiangbei Hospital, Nanjing, Jiangsu, China

[2]Zhangjiang Center for Translational Medicine, Shanghai Biotecan Pharmaceuticals Co., Ltd., Shanghai, China

[3]Teaching Research Department, Changji Branch of the First Affiliated Hospital of Xinjiang Medical University, Changji, Xinjiang, China

*CORRESPONDENCE

Huihui Jiang

hhjiang16@fudan.edu.cn

Xiangming Zhang

506303415@qq.com

Jiaying Li

641557266@qq.com

[†]Siliang Xia, Yun Liu, and Mengzhu Wang contributed equally to this work.

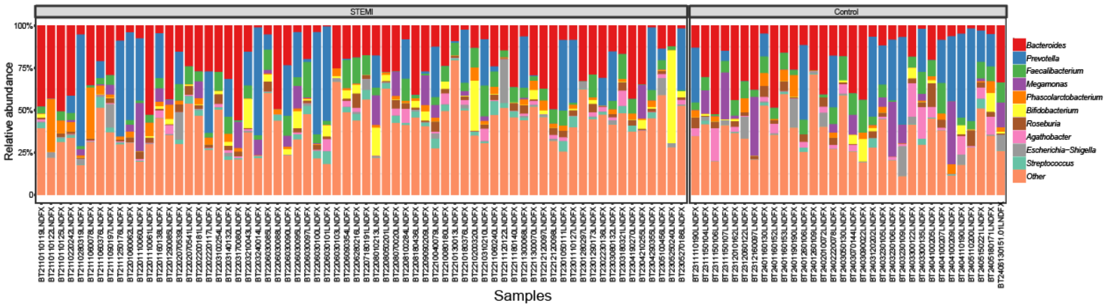

SUPPLEMENTARY FIGURE 1

Composition and distribution of gut microbiota at the genus level in male individuals without coronary heart disease (Control, n=32) and male patients with ST-segment elevation myocardial infarction (STEMI, n=66). The ten most prevalent genera with the highest relative abundance were represented in distinct colors, while those with lower abundance were collectively categorized as 'other.'

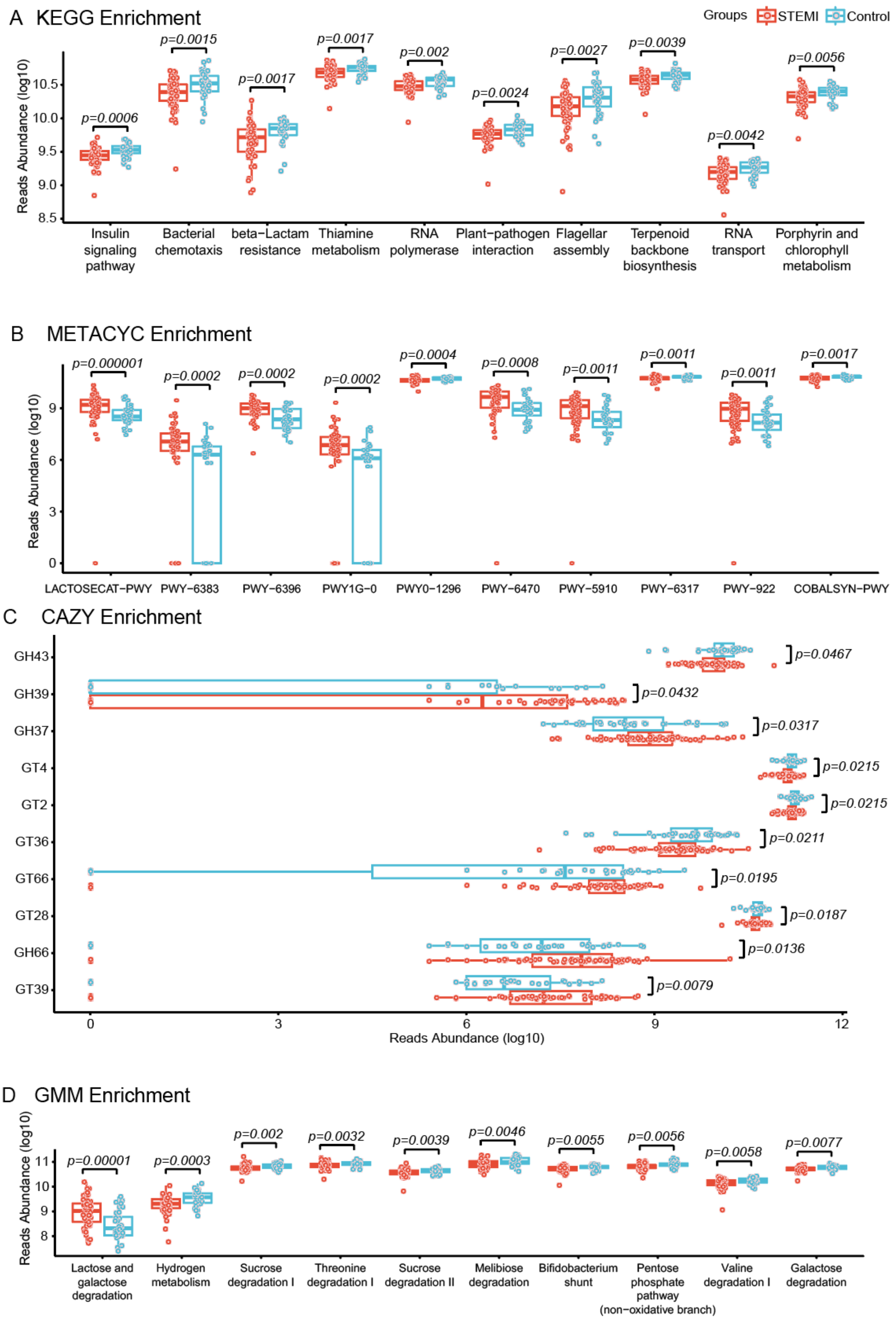

SUPPLEMENTARY FIGURE 2

PICRUSt2-based functional prediction analysis was conducted to identify enriched metabolic pathways distinguishing the control group from the STEMI group, encompassing KEGG, METACYC, CAZY, and GMM databases. The top 10 differentially enriched pathways within KEGG (A), METACYC (B), CAZY (C), and GMM (D) between these two

groups are presented.

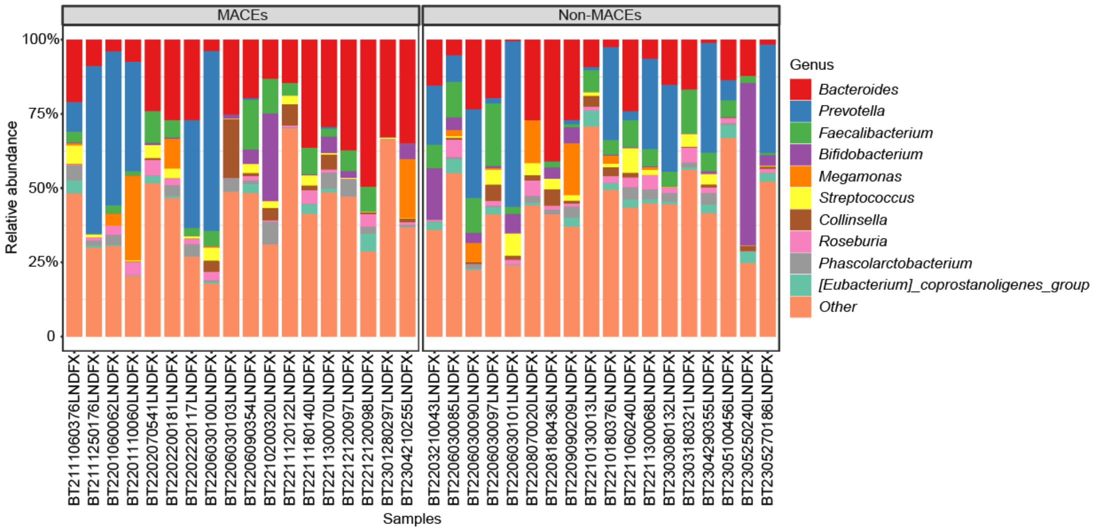

SUPPLEMENTARY FIGURE 3

The composition and distribution of gut microbiota at the genus level were analyzed in patients with STEMI who experienced major adverse cardiovascular events (MACEs, n=18) and age-matched patients with STEMI without MACEs (Non-MACEs, n=18). The top 10 genera with the highest relative abundance were illustrated in distinct colors, while those with lower abundance were categorized as 'other.'

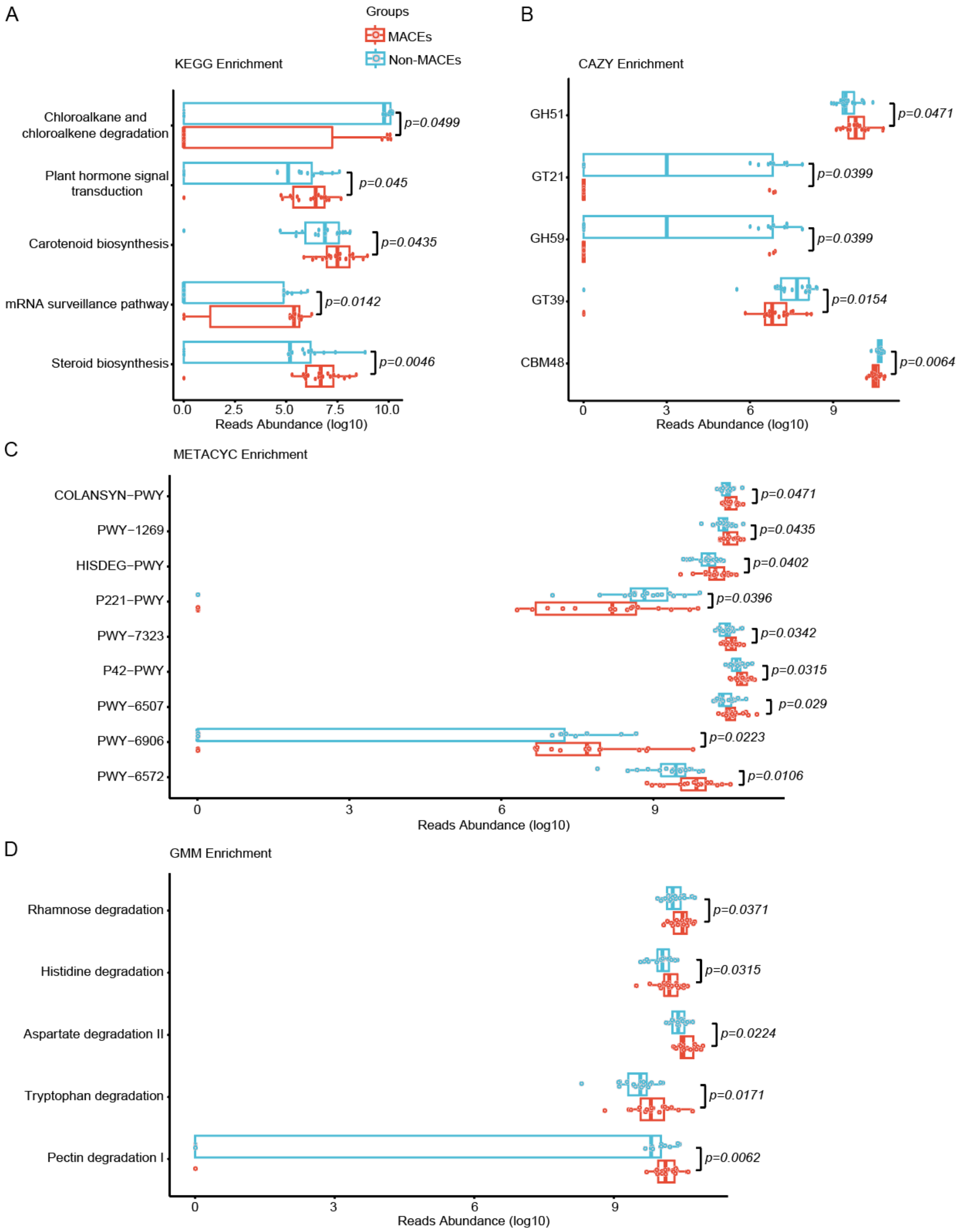

SUPPLEMENTARY FIGURE 4

Picrust2 functional predictive analysis was conducted to identify enriched metabolic pathways distinguishing the Non-MACEs group from the MACEs group, encompassing KEGG, CAZY, METACYC, and GMM. The top 5, 5, 9, and 5 differentially expressed signaling pathways in KEGG (A), CAZY (B), METACYC (C), and GMM (D) between these two groups were highlighted, respectively.
